# Extremely large area (88 mm × 88 mm) superconducting integrated circuit (ELASIC)

**DOI:** 10.1038/s41598-023-39032-6

**Published:** 2023-07-21

**Authors:** Rabindra N. Das, Vladimir Bolkhovsky, Alex Wynn, Jeffrey Birenbaum, Evan Golden, Ravi Rastogi, Scott Zarr, Brian Tyrrell, Leonard M. Johnson, Mollie E. Schwartz, Jonilyn L. Yoder, Paul W. Juodawlkis

**Affiliations:** Quantum Information and Integrated Nanosystems Group, MIT Lincoln Laboratory, 244 Wood Street, Lexington, MA 02421 USA

**Keywords:** Nanoscale devices, Techniques and instrumentation

## Abstract

Superconducting integrated circuit is a promising “beyond-CMOS” device technology enables speed-of-light, nearly lossless communications to advance cryogenic (4 K or lower) computing. However, the lack of large-area superconducting IC has hindered the development of scalable practical systems. Herein, we describe a novel approach to interconnect 16 high-resolution deep UV (DUV EX4, 248 nm lithography) full reticle circuits to fabricate an extremely large (88 mm × 88 mm) area superconducting integrated circuit (ELASIC). The fabrication process starts by interconnecting four high-resolution DUV EX4 (22 mm × 22 mm) full reticles using a single large-field (44 mm × 44 mm) I-line (365 nm lithography) reticle, followed by I-line reticle stitching at the boundaries of 44 mm × 44 mm fields to fabricate the complete ELASIC field (88 mm × 88 mm). The ELASIC demonstrated a 2X–12X reduction in circuit features and maintained high-stitched line superconducting critical currents. We examined quantum flux parametron circuits to demonstrate the viability of common active components used for data buffering and transmission. Considering that no stitching requirement for high-resolution EX4 DUV reticles is employed, the present fabrication process has the potential to advance the scaling of superconducting qubits and other tri-layer junction-based devices.

## Introduction

Superconducting integrated circuits (SIC), such as single-flux-quantum-based (SFQ) digital integrated circuits^1^, use Josephson junctions (JJs) as switching devices with an extremely high switching speed (~ 1 ps), ultralow switching power (~ 2 aJ/bit), and nearly lossless signal propagation to encode, process, and transport data with a significantly increased clock rate (10×) and power efficiency (100×) relative to advanced-node CMOS at scale^2^. The existing SIC technology is practically limited to 10 mm × 10 mm, or more typically, 5 mm × 5 mm, for single-chip systems. However, because of their relatively low integration scale, systems based on superconducting technology require a large number of interconnected SIC chips for practical applications, and a scalable approach is necessary to achieve this goal.

The maximum size of a superconducting integrated circuit (SIC) chip is typically limited by the reticle area of the lithographic stepper tool used to pattern the integrated circuit. This limitation is compounded for superconducting integrated circuits (SICs), in which the basic switching element, the Josephson Junctions (JJs), is orders of magnitude larger than state-of-the-art CMOS transistors. High-density JJs fabrication processes have produced chips with a maximum area^3^ of ~ 100 mm^2^ and maximum circuit densities^4^ of 7.4 × 10^6^ JJ/cm^2^ which limits the total number of JJs in a large (22 mm × 22 mm) reticle to 3.5 × 10^7^ in an ideal case. Methods to increase the number of JJs in a SIC beyond this limit include the introduction of niobium nitride (NbN) to increase kinetic inductance^5^, niobium titanium nitride (NbTiN) with a short coherence length (~ 5 nm) for narrow line widths and shorter wire lengths for high-density circuits^6^, introduction of higher J_c_ JJ layers, and introduction of multiple JJ layers within the process stack^4^. The challenges of applying these methods include composition variation^7^ for multicomponent systems, limited critical current densities, variability of inductors and JJs, and mutual inductances leading to low isolation^8,9^. In addition to increasing the circuit density at the chip level, we propose the integration of a number of high-density SIC by flip-chip interconnection in a large format, but with a lower-density active chip carrier, the ELASIC. We believe that the ELASIC concept provides a significant advance in SIC scaling and has the potential to transform a wide variety of SIC applications, including sensors^10–13^, cryogenic digital control^14–18^ circuits, amplifiers^19,20^, and classical cryogenic computing^21–23^. Superconducting multi-layer circuit^24^, a passive chip carrier, technology is the key to building a scalable superconducting system owing to its large area of integration and the ability to preselect and rework individual component chips (chiplets) within the carrier, bypassing single-chip yield constraints.

MIT Lincoln Laboratory (MIT LL) has developed several passive superconducting circuit-based chip carrier fabrication and integration processes^24–32^ for cryogenic computing. For example, passive large-area superconducting circuits (chip carriers) with low passive transmission line (PTL) losses afforded by superconducting materials enable a record-high 10 GHz serial chip-to-chip communication bandwidth covering a distance of over a meter^28^. The technology further demonstrated^28^ synchronous communications between eight superconducting Reciprocal Quantum Logic (RQL) chips powered by a passive large-area (32 mm × 32 mm) circuit with a resonant clock distribution network at a data rate of up to 8 GHz with 3 fJ/bit dissipation. Furthermore, the passive superconducting circuit technology extended^29^ isochronous data links across RQL-passive carrier-superconducting (niobium) flex operated with a clock margin of 3 dB @ 3.6 GHz with 5 fJ/bit dissipation. Heterogeneous integration^30–32^ used various superconducting interconnect materials for microbumps to integrate up to 20 mm × 20 mm SICs. In all these cases, a large-area (32 mm × 32 mm ) passive superconducting circuit-based chip carrier was used for chip-to-chip communications, and the PTL-based interconnection potentially limits their ability to provide sufficient bandwidth and low latency for next-generation superconducting electronics. A large-area active superconducting chip carrier with a Josephson transmission line (JTL), passive transmission line (PTL), and driver-receiver circuits to distribute information without loss between superconducting integrated circuits is highly desirable for realizing complex hybrid computing architectures. However, this has yet to be demonstrated. In this paper, we present an implementation of such a large cryogenic active superconducting chip carrier.

This study demonstrates an active chip carrier known as an extremely large-area superconducting integrated circuit (ELASIC), fabricated by interconnecting 16 EX4 reticles with larger field-size I-line reticles. A traditional stitching approach for interconnecting 16 reticles uses 24 stitch boundaries between Deep UV EX4 reticles per layer; this large number of interconnect masks increases the number and complexity of process steps relative to our approach, resulting in a significant risk of yield loss and limiting design flexibility. In this study, we used a novel fabrication technique for implementing ELASIC with the intent of achieving maximal flexibility and complexity with minimum yield loss. ELASIC (active carrier) fabrication uses Deep UV EX4 reticles (22 mm × 22 mm) to create individual building blocks interconnected by four EX4 reticles with larger field i-line reticles (44 mm × 44 mm), followed by i-line reticle stitching to fabricate a full ELASIC field of 88 mm × 88 mm (7744 mm^2^). We used a high-resolution DUV stepper (Canon EX4 reticles) for the junction layer and a large-field i-line (365 nm) stepper for the interconnection and stitching. Reticle stitching is ubiquitous in the semiconductor industry in high-performance computing applications. TSMC reported 2500 mm^2^ stitched passive interposer circuits^33,34^ for chip-on-wafer-on-substrate-based multi-chip integration technology for high-performance computing (HPC). Although reticle stitching is not new, interconnecting 16 high-resolution DUV stepper (Canon EX4) reticles (7744 mm^2^) without the introduction of stitching at individual EX4 reticles, instead of stitching with a large-field i-line reticle, as a method to significantly reduce the number of fabrication steps and improve yield, has not been demonstrated before.

## Results

An ELASIC is a large (88 mm × 88 mm) active superconducting chip carrier technology that leverages standard SFQ5ee^35–37^ processes to integrate junction devices into the chip carrier, featuring active and passive superconducting transmission lines, driver-receiver circuits to distribute information without loss of signal integrity between widely spaced integrated circuits, and the potential for data buffering or memory within the ELASIC. The development of ELASIC for integrating a large number of superconducting chips into a single system could have impacts on a range of important technological areas. This approach improves the functionality (number of JJs, latency, and bandwidth) within a single interconnected system, enabling larger and more capable system designs. Additionally, the ELASIC platform, when used as an active chip carrier, enables the integration of a wide variety of cryogenic components^24–32^. This enables system designers to move buffering, synchronization, local caches, or other standard circuit elements to the ELASIC for chip-to-chip communication. In contrast to the power-driven need for closer integration of components within CMOS systems, SCE circuits are driven less by power constraints but are equally or more sensitive to interconnect latency owing to high operating frequencies. Additionally, increasing the integration scale through packaging is a larger driver of SCE, for which on-carrier memory offers a significant benefit. By adding an active junction layer within the carrier, this approach favors active-to-active bonding with short distances between active elements such as logic and memory (see Supplemental Materials S1), enabling higher bandwidth and lower latency communications than an equivalent active-to-passive platform^28,29^.

Previous studies at MIT LL on passive superconducting chip carrier fabrication used I-line (365 nm) lithography and reticle stitching. However, the addition of a Josephson junction layer to the chip carrier interconnect layer requires high-resolution deep UV (DUV) EX4 (248 nm) lithography to ensure sufficiently low process variability. Component variation with standard deviations below approximately 5% is desirable to achieve high yield and low timing variation at an integration scale greater than 10^6^ Junctions^35–37^. Beyond the addition of active transmission lines and amplifiers, active chip carrier functional circuits can be designed using Josephson junctions, inductors, resistors, transformers, and transmission lines that are compatible with the requirement of less than 5% variation in key parametric margins (critical current, self-and mutual inductance, and resistance). ELASIC offers several advantages over conventional passive superconducting chip carriers.

- Junction devices were integrated into the passive chip carrier to increase functionality (i.e., active transmission lines, drivers, receivers, repeaters, transformers, amplifiers, etc.).
- Reduced feature size (2.2X smaller line width than passive chip carrier^28,29^)
- Reduce via diameter (6X smaller than passive chip carrier^28,29^)
- Smaller resistor (2X smaller resistor width and via diameter than passive chip carrier^28,29^)
- Use deep UV lithography for microbump fabrication (see Supplemental Materials S1) with reduced micro-bump pitch (2X–12X smaller micro-bump pitch than passive chip carrier^30–32^)

**Figure 1:**
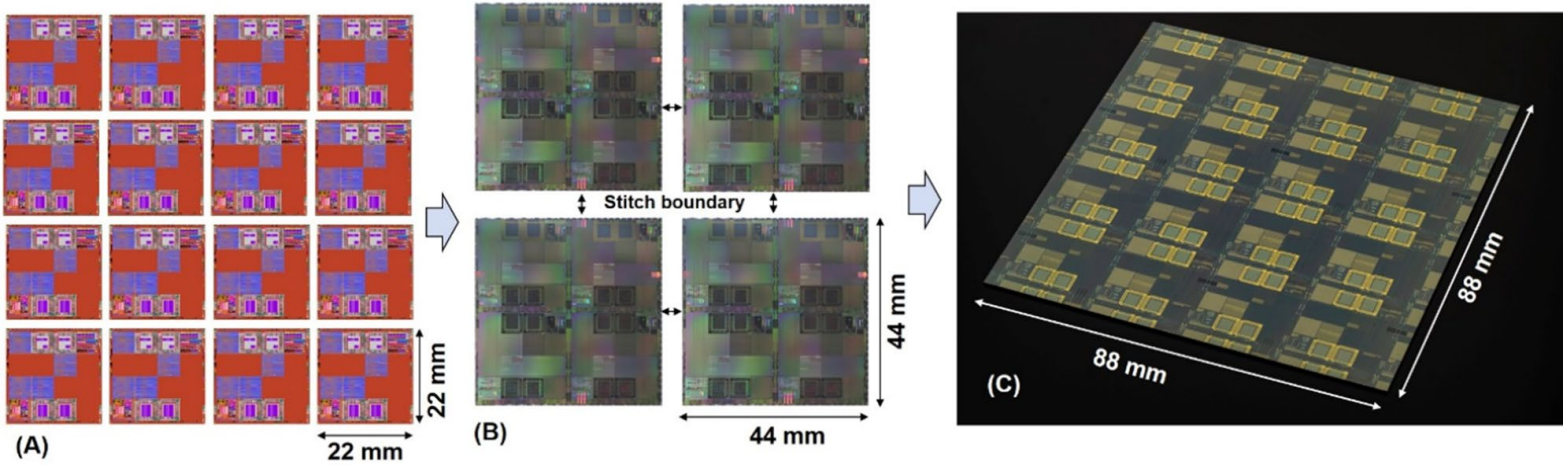
ELASIC fabrication process flow (**A**) 16 individual as designed GDS image of 22 mm × 22 mm deep UV (DUV) EX4 reticle building blocks, (**B**) As fabricated optical image of 44 mm × 44 mm I-line reticle interconnecting 4 EX4 DUV reticles, and (**C**) As fabricated optical image of extremely large area (88 mm × 88 mm) superconducting integrated circuit (ELASIC) interconnecting 16 DUV Canon EX4 reticles. Four EX4 reticle based circuit layers are interconnected with an I-line reticle based circuit layer using sub-micron vias. Four I-line reticles (as shown in **B**) are connected via stitching, ultimately allowing full connectivity of 16 EX4 reticles through the I-line circuit layer to create ELASIC.

Our interconnection approach to join 16 Canon EX4 DUV reticles is enabled by first interconnecting four EX4 DUV reticles using large-field I-line reticles, connecting adjacent blocks of four EX4 reticles with one I-line mask per block, followed by four additional I-line stitching masks to interconnect the four blocks along their edges. Figure 1 shows the GDS layout artwork and optical images of the key steps during interconnection with I-line reticles, which include EX4 DUV reticles for junction layers, interconnection of four EX4 DUV reticles with a single large-format I-line reticle, and reticle stitching to create a complete 88 mm × 88 mm ELASIC. The design consists of 16 identical reticles interconnected using i-line reticles, with each reticle performing as an individual computing module with the same or different functions. The EX4 DUV reticle contains JJs, which are the basic functional active elements of the ELASIC. The I-line reticle has DC and microwave lines to interconnect individual EX4 DUV reticles, with superconducting niobium (Nb) vias providing connectivity between the two reticles (DUV to I-line). The I-line reticle uses a stitching process to extend the active Josephson junction functionalities and wire routing to the entire 88 mm × 88 mm field. The traditional approach for interconnecting 16 reticles involves stitching between the reticle boundaries^31,32^. This approach requires 24 stitch boundaries between the reticles per layer, and has a significant risk of yield loss and limited flexibility. The yield and cost are both proportional to the number of masks and the processing steps. Therefore, it is highly desirable to reduce these values to a minimum. The current fabrication approach has two significant advantages over the traditional approaches. First, the approach enables the extension of narrow linewidths and low variability achievable with EX4 DUV lithography to a large-format 88 mm × 88 mm field, without individual DUV reticle stitching. This is particularly advantageous for simplifying the fabrication process, which is expected to improve the yield, and is typically a limiting factor for large-format ICs. Process simplification can be achieved because deep UV (DUV) lithography with a small overlay (~ 50 nm) can be used where necessary on JJ-based building blocks within the reticle, and less precise I-line lithography with an overlay of less than 100 nm can be used for the remaining passive interconnections between the reticles. Therefore, the mixing of I-line lithography with DUV allows for manageable stitching along with accurate features of the key layers. Second, ELASIC has a surface compatible with microbump fabrication^28–32^ which provides the flexibility to use 2-stack and 3-stack integration with known good dies for heterogeneous integration. Furthermore, microbumps on the ELASIC allow superconducting flex integration^29^ to distribute signals between ELASICs within a cryogenic system (mK to 4 K). Deep UV reticles further help to create small microbumps (see Supplemental Materials S1), enabling a chip-like wiring density.

**Figure 2:**
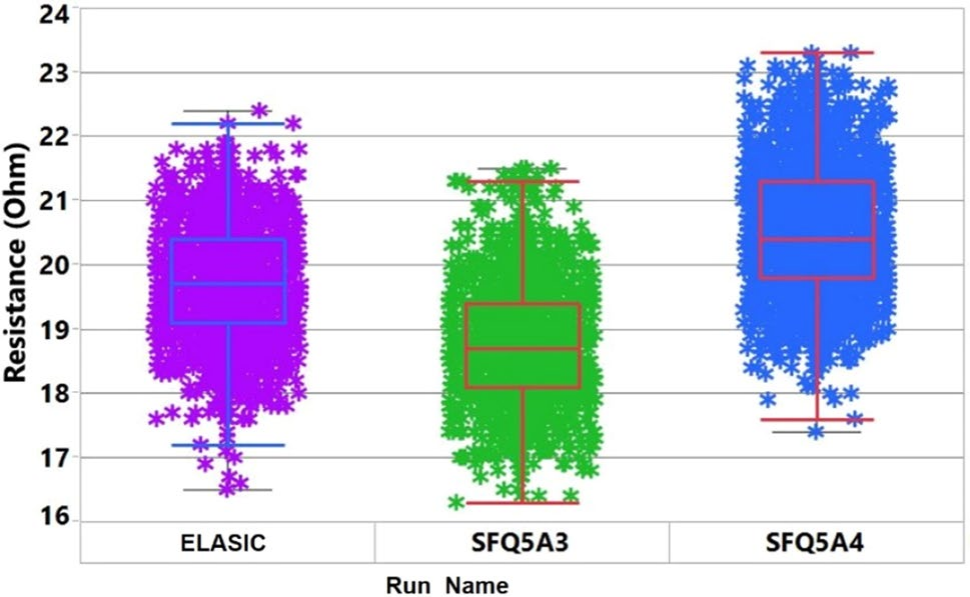
Comparison of room-temperature Junction resistance variability data for the ELASIC platform and MIT LL’s standard DUV-reticle-based SIC fabrication processes (SFQ5A3, SF5A4).

We characterized an 88 mm × 88 mm ELIAC at room temperature using an automated wafer probe. Approximately 150 test structures were measured for rapid feedback (Supplemental Materials S2) regarding the new fabrication process. We compared the results with the MIT LL standard SFQ5ee^35–37^ fabrication process, which uses all the DUV reticles to gauge the parametric variation and yield. Figure 2 shows representative results of the 1 µm Cross Bridge Kelvin Resistance (CBKR) junction resistance across the wafer for various SFQ5ee runs and compares it with the new combined DUV-I-line (ELASIC) fabrication process. The JJ resistance across the wafer for the previous SFQ5ee runs is comparable to that of the new approach, which indicates that the new fabrication approach to implementing the ELASIC platform has a comparable junction uniformity. The normal resistance, R_n_, measured at room temperature is highly correlated with the critical current, I_c_, measured at 4 K. This is characterized by the Ambegaokar-Baratoff^38^ relationship and a relatively uniform I_c_R_n_ product for a given superconductor process. Although recently developed systems may enable wafer probing directly at 4 K in the future^39^, normal resistance measurements at room temperature are preferable compared to direct I_c_ measurements to characterize the fabrication process because room-temperature wafer-scale automated probers can be used to to collect large statistics on individual junctions.

**Figure 3:**
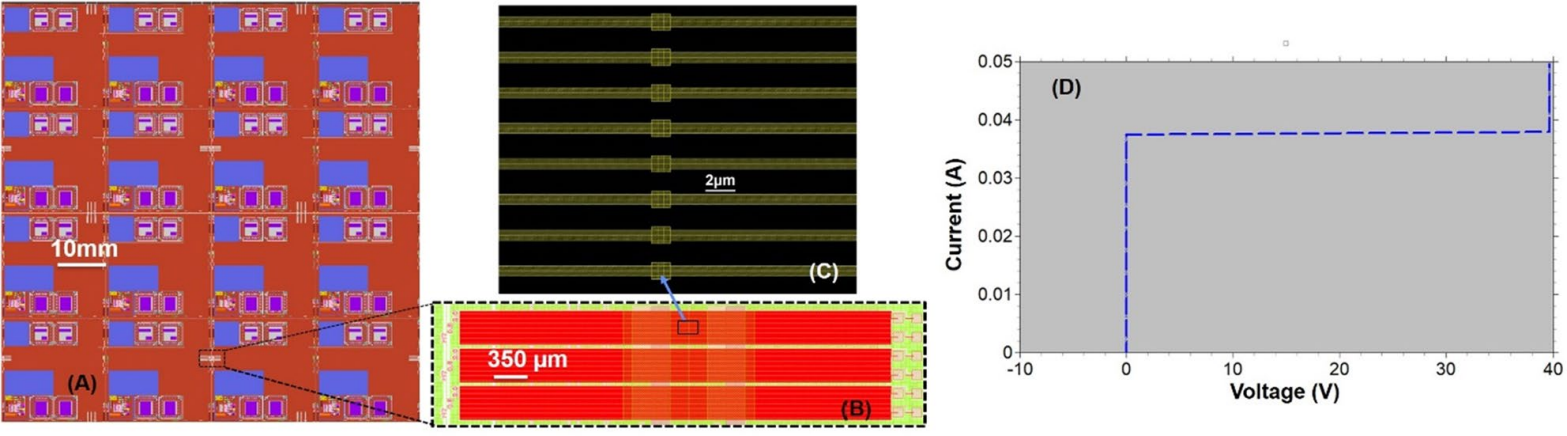
(**A**) ELASIC GDS image, (**B**) 0.8 µm stitched snake & combs lines, (**C**) Enlarged view of 0.8 µm stitched snake & combs lines at the stitch boundary. Stitched line of 0.8 µm linewidth showed a reduction of approximately 70–110 nm within a 1 µm region of the overlap^32^. To make the stitching process manufacturable, we impose a design rule for stitched wires with a width of 1.5 µm or narrower to increase local linewidth by 0.25 µm on each side of the stitch boundary within a 1 µm long overlap region. This additional region of increased linewidth compensates for the post-fabrication reduction at the overlap, in addition to compensating for potential misalignment, to maintain a high critical current with negligible impedance impact below 1 THz^31^. (**D**) Measured critical current (I_c_) of a 0.8 µm stitched snake and comb Nb lines at 4.2 K. An apparent series resistance on the order of 45 mΩ is due to the final connection to the test structures being made by a single wire bond path, which contributes an additional series resistance to the test structure equal to the resistance of the wire bond.

An ELASIC was attached to a printed circuit board (PCB) and wire bonded to enable measurement of the I–V characteristics of 0.8 µm-linewidth stitched “snake and combs” test structures at 4.2 K. Figures 3A,B show 0.8 µm stitched snake and comb lines going back and forth across the stitch boundary. We measured multiple 0.8 µm stitched snake and comb lines, going back and forth for approximately 20 times in a series of 5 mm wire lengths, which had critical currents in the range of 30–40 mA at 4.2 K, which is comparable to typical measurements obtained in the SFQ5ee process. Figure 3D shows the I–V curve of the 0.8 µm snake/combs stitched line as a representative example. From the I–V data shown in (Fig. 3C) the Nb-stitched lines had a critical current of approximately 38 mA at 4.2 K. The large number of stitch boundaries for a long narrow line with 0.8 µm width and 2 µm space, and the high critical current/high current-carrying capacity of the stitched Nb lines make this process suitable for building extremely large-area integrated circuits (ELASICs) and capable of sustaining interconnect requirements for superconducting computing systems for heterogeneous integration.

**Figure 4:**
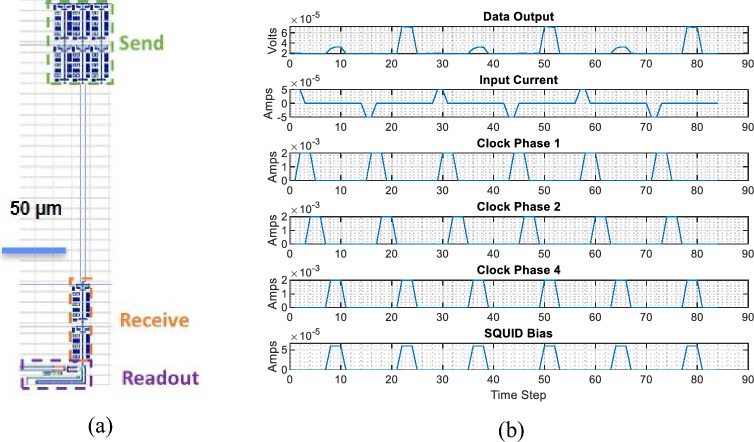
Demonstration of QFP data transmission at 4.2 K. (**a**) Layout of a QFP circuit composed of two inverters to transmit the input data, followed by a 200-µm-long interconnect line with two receiving inverters and a final DC SQUID that is used for readout of the final QFP state (**b**) Measurement results show operation of all elements, including correct data transmission between pairs of inverters across the interconnect line. Timesteps are approximately 1 ms, but are not locked to an external clock source, and are set in practice by communication with the data acquisition system.

We characterized the quantum flux parametron (QFP) circuits at cryogenic temperatures to validate the fabrication process. Ultra-low-energy superconducting logic gates based on QFPs^40–42^ are promising, in part owing to the use of identical unit cells with relatively wide parametric operating margins. QFP data transmission was used to test the process, which is limited in communication distance by the inductance between cells, and therefore requires multiple send/receive inverters or buffer pairs on large-area circuits to cover the distance between circuit elements. The objective of this measurement was to test the operation of the QFP inverters fabricated in this process, demonstrate the use of inverter pairs to send and receive data across an on-chip interconnect line, validate the continuity of superconductivity across the stitch boundary because of the circuit’s inability to tolerate any series resistance at the measured frequencies (kHz), and demonstrate the operation of functional circuit blocks that are expected to be commonly used in ELASIC configurations. The QFP circuit generates a larger output current in response to a small input current flowing into the QFP during the clock arrival. This mechanism reamplifies data at each inverter or buffer stage. Figure 4 shows an example of a QFP inverter in series, demonstrating the data communication across two pairs of inverters.

**Figure 5:**
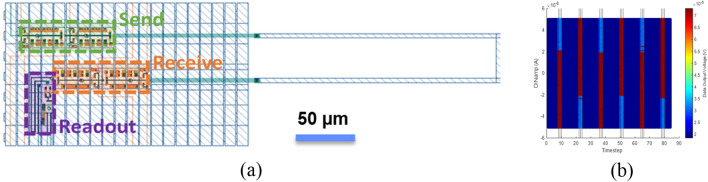
(**a**) Layout of a QFP driver-receiver circuit, composed of two pairs of QFP inverters, one acting as a driver and one acting as a receiver, followed by a DC-SQUID for isolation and amplification of the QFP data-level signal (~ 5 µA) to a ~ 50 µV output signal. (**b**) Measurement results show a consistent DC offset from readout SQUID in the data output, with output data patterns matching the transmitted data pattern (1, 0, 1, 0, 1, 0), indicating a successful transmission and correct circuit operation. A tendency to skew towards positive values in the output data pattern can be seen as overlapping red bars (data “1”) in a plot of readout SQUID voltage for data levels below about 2 µA.

Various test circuits are designed to demonstrate the benefits of our fabrication scheme. Test circuits composed of pairs of identical QFP inverters as driver and receiver elements, with strip-line and microstrip connections between active elements, were fabricated and tested to evaluate circuit functionality and evaluate data transmission across stitch boundaries. An example layout is shown in Fig. 5 along with the measured results for structures separated by a transmission distance of 200 µm. Overall, circuits functioned as designed for a typical QFP data signal level of 5–10 µA; minor DC offsets limited the minimum transmissible current amplitude to approximately 2 µA, equivalent to roughly a 300 pH transmission inductor for Φ_0_·0.3, where 0.3 is the approximate coupling constant of the mutual inductor at the output of an inverter.

Consistent with this DC-offset-limited communication, driver-receiver pairs functioned for test structures with 200 µm spacing or inductances below 100 pH and failed for connections with inductances above 200 pH. From tests of similar structures in the SFQ5ee process^35–37^, it is anticipated that the addition of a third inverter element in the driver and receiver circuits would reduce the DC overlap region and would improve the maximum transmission inductance. Additionally, we note that successful operation of these inverters at frequencies below 1 kHz implies an upper bound of the series resistance in the stitch boundary of about 2 µΩ, calculated by solving for the resistance necessary to dissipate I_c_·Φ_0_ ~ 10^−19^ J in 3 ms (300 Hz testing speed), with a nominal 15 µA output current amplitude crossing the stitch boundary.

## Discussion

In summary, we demonstrated a new approach for fabricating extremely large-area superconducting integrated circuits (ELASICs) on a 200 mm-diameter silicon wafer interconnecting 16 high-resolution DUV (248 nm lithography) reticles (each of 22 mm × 22 mm) into a large superconducting system. Our approach uses I-line (365 nm lithography) reticle stitching with only four stitch boundaries per layer to interconnect 16 DUV reticles. This helps minimize the yield loss, which is roughly proportional to the number of mask steps, and consequently allows for the creation of larger format systems. We believe that this is the first demonstration of such a large (88 mm × 88 mm) superconducting integrated circuit produced by interconnecting high-resolution DUV reticles without stitching individual reticles.

The ELASIC provides a platform for interconnecting a large number of discrete SICs. Room-temperature electrical measurements indicated that the present fabrication approach is comparable to standard SICs fabricated using traditional EX4 DUV lithography, and maintains run-to-run consistency with unstitched circuits fabricated using the MIT LL SFQ5ee process. The ELASIC-stitched I-line test structures exhibited critical currents in the range of 30–40 mA at 4.2 K for 0.8 µm-wide stitched lines. Cryogenic measurements of the QFP circuits showed data transmission through the QFP blocks, further supporting the ELASIC fabrication approach. The new fabrication approach is capable of transmitting QFP data signals in the range 5–10 µA. Overall, we developed a versatile interconnection approach to fabricate very large superconducting integrated circuits for heterogeneous integration suitable for computing scalability beyond arrays of a few chips. The ELASIC fabrication process has achieved an important milestone towards large-area active superconducting circuit fabrication and has the potential to advance the scaling of superconducting qubits^43^ and other tri-layer Josephson junction based devices^15^.

We believe that the current interconnection scheme can be extended to CMOS circuits for fabricating large-area active interposers. High-performance exascale computing^44^ uses an active interposer for active-to-active bonding, which is necessary to increase the bandwidth and reduce latency. The current approach for creating large-area active interposers can overcome the existing interposer size limitations for advanced high-performance computing^45^.

## Methods

**Figure 6:**
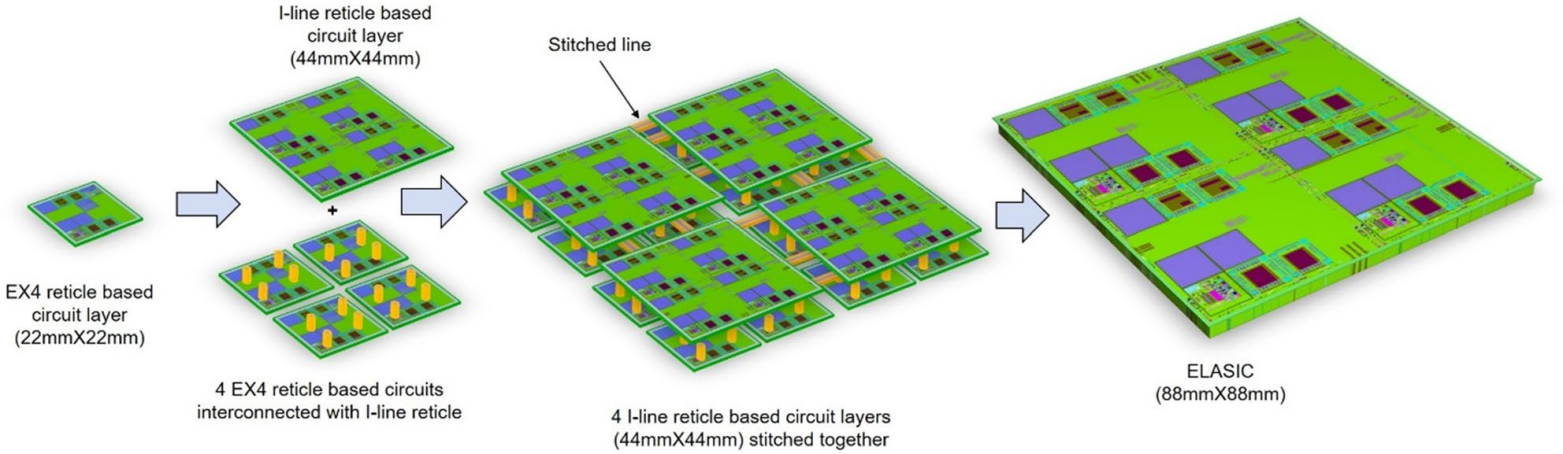
3D view of the ELASIC (active superconducting chip carrier) fabrication process starting from individual EX4 DUV reticle based circuits and their interconnection schemes to fabricate the ELASIC. Four EX4 DUV reticle based circuit layers (22 mm × 22 mm)are interconnected with an I-line reticle based circuit layer (44 mm × 44 mm) using sub-micron EX4 DUV vias (yellow). I line reticles (44 mm × 44 mm) are connected via stitching, ultimately allowing full connectivity of the 16 EX4 DUV reticles through the I-line circuit layer.

To fabricate the ELASIC, we utilized the Lincoln Laboratory’s SFQ5ee^35–37^ process to fabricate niobium-based integrated circuits using Nb/Al–AlO_x_/Nb tri-layer Josephson junctions with a J_c_ of 10 kA/cm^2^ and junction diameters down to 500 nm. This process utilizes high-resolution deep UV (248-nm photolithography) for multilayer Nb wiring with minimum circuit feature sizes down to 350 nm, Mo-based shunt resistors, and Nb-based superconducting via interconnects between all metal layers separated by a silica-based dielectric. Figures 1, 6 show the design schemes for ELASIC and the corresponding images of the fabricated devices. We use a 200 mm wafer fabrication process consisting of 13 photomasks:8 (tiled) EX4 DUV photomasks with a 22 mm × 22 mm field size were used to create junctions, resistors, interconnects, etc., and 4 (tiled) I-line photomasks with a field size of 44 mm × 44 mm were used to interconnect the DUV reticles and reticle stitching. Circuit wiring on individual I-line reticles with a field size of 44 mm × 44 mm was stitched/joined^32^ together at a stitch boundary to create an ELASIC field of 88 mm × 88 mm. In summary, four EX4 DUV reticles were interconnected with each other using a single I-line reticle, and four I-line reticles used reticle stitching to interconnect with each other to create a complete ELASIC from 16 interconnected EX4 DUV reticles. The detailed stitching process has been described in our previous paper^32^.

**Figure 7:**
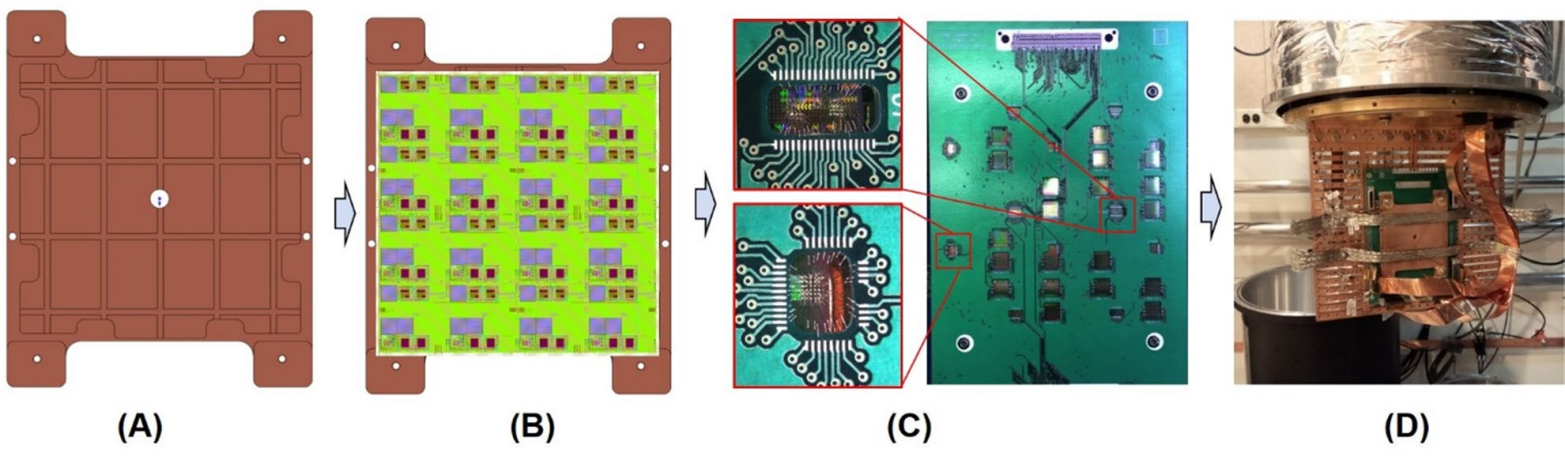
ELASIC packaging process flow (**A**) Copper plate (1.4 lb). (**B**) ELASIC attached to copper plate. Thermal grease and silver paint were used to attach ELASIC to copper plate. (**C**) Placement of PCB, wirebonds and ardent connectors interface on cryocooler motherboard. (**D**) Mount assembly to cryo-cooler for thermal cycling.

Individual test structures were initially tested in liquid He to evaluate the fabrication approach. The ELASIC sample was then diced to 88 mm × 88 mm and mounted on a custom copper plate using silver paint and Apiezon N grease. A custom FR4 printed circuit board (PCB) was attached to the copper plate with screws, and wire-bond connections were made from the PCB to the silicon sample below (achieved with cutouts in the PCB to allow access to the silicon part below). The packaged sample assembly was mounted on a custom motherboard printed circuit board (PCB) mounted on a 4 K cryocooler. A total of 300 signals were passed to the motherboard via two Ardent compression mount connectors and carried to room temperature on six 51-pin micro-d flex cables. The cryocooler was equipped with a high-permeability shield, and the materials used near the sample (including PCBs, connectors, and cables) were carefully selected to avoid any residual magnetic field. A full ELASIC (88 mm × 88 mm) sample was assembled as shown in Fig. 7. The ELASIC devices were thermally cycled multiple times in a cryocooler to check their integration stability, wiring reliability, and I–V characteristics.

### Characterization

Details of the various characterization methods are provided in the supplementary section (S1).

### Measurements

Detailed room-temperature measurement data from the wafer probe are provided in supplementary section (S2).

## Supplementary Information

Supplementary Information 1.

Supplementary Information 2.

Supplementary Information 3.

Supplementary Information 4.

## Data Availability

The datasets used and/or analysed during the current study available from the corresponding author on reasonable request.
