# Supplementary material for: Extremely large area (88 mm × 88 mm) superconducting integrated circuit (ELASIC)
Source: Sci Rep. 2023 Jul 21;13:11796. doi: 10.1038/s41598-023-39032-6 (PMC10361992; doi:10.1038/s41598-023-39032-6)
Supplement: Supplementary file 1 — Supplementary Information 1. [file 41598_2023_39032_MOESM1_ESM.docx]

**Extremely Large Area (88 mm X 88 mm) Superconducting Integrated Circuit (ELASIC)**

Rabindra N. Das^*^, Vladimir Bolkhovsky, Alex Wynn, Jeffrey Birenbaum, Evan Golden, Ravi Rastogi, Scott Zarr, Brian Tyrrell, Leonard M. Johnson, Mollie E. Schwartz, Jonilyn L. Yoder and Paul W. Juodawlkis

Quantum Information and Integrated Nanosystems Group

MIT Lincoln Laboratory

244 wood street, Lexington, MA02421

[Rabindra.das@ll.mit.edu](mailto:Rabindra.das@ll.mit.edu)

Supplementary Materials

Sections S1-S2

Figs. S1 to S5

Tables S1

# **S1: ELASIC Characterization**


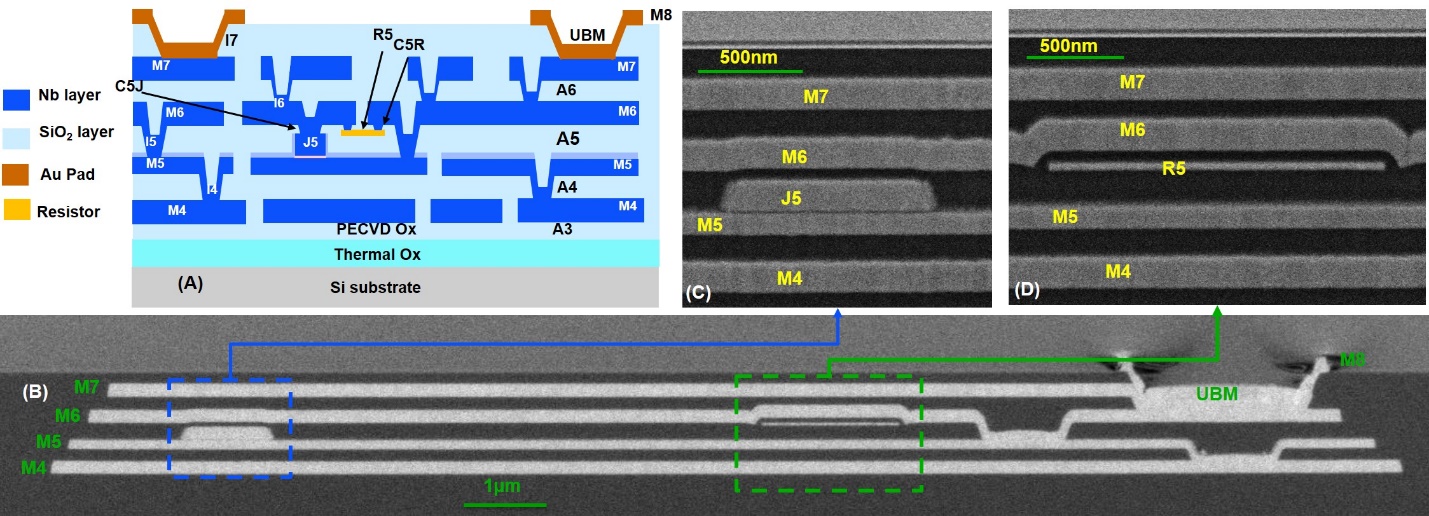


| **Figure S1:** (A) Schematic of Extremely large area superconducting integrated circuit (ELASIC) cross-sectional view, (B) corresponding FIB SEM cross-sectional view, (C)-(D) Enlarged SEM view of the ELASIC. |
| --- |

The ELASIC fabrication used Nb/Al-AlO_x_/Nb trilayer JJs. The use of ELASIC technology allows a combination of deep submicron niobium Josephson junction-based functional circuits^36^ and multilayer passive interconnect-based circuits for routing signals from the SIC to external connections. Connections between the superconducting integrated circuit (SIC) and ELASIC are achieved using an indium bump-bonding process^28-32^ that is fully compatible with JJ-based electronics. Multiple passive wiring layers in the ELASIC allow a large number of connections to be made for the superconducting chip while maintaining appropriate shielding and isolation to suppress crosstalk. As an example, a cross-sectional view (schematic and FIB SEM) of a large (88 mm × 88 mm) ELASIC is shown in **Figure S1**. It has five Nb metal layers, one junction layer, and one resistor layer interconnected using a Nb via. Sixteen EX4 reticles, each of which was 22 x 22 mm^2^, were interconnected to a large (88 mm × 88 mm) ELASIC. All the layers below M6 used DUV masks. We created all vias including I5, C5J, and C5R using DUV reticles, which is critical for maintaining the feature sizes associated with the DUV features on adjacent layers without requiring substantially larger via surrounds, which would have implications for circuit density and impedances. Circuits on M6 and above primarily use impedance-controlled lines to connect individual EX4 features. Nb vias were used for both DUV and I-line reticles for interconnection.

The EX4 reticle circuits of the ELASIC were fabricated on 200-mm-diameter silicon wafers using MIT LL’s SFQ5ee process^35-37^, a niobium-based superconducting integrated-circuit fabrication process appropriate for integrating superconductors, semiconductors, and photonic chips. The fabrication supports a Nb/Al-AlO_x_/Nb Josephson junction trilayer with a J_c_ of 10 kA/cm^2^ and Nb^35-37^ wiring layers separated by a PECVD silicon oxide dielectric and Nb vias used to interconnect metal layers to create superconducting circuits. The circuit has bump metal pads, composed of 20 nm Ti (adhesion layer), 50 nm Pt (barrier layer), and 150 nm Au, for flip-chip integration. The target thicknesses of all layers of the ELASIC using the SFQ5ee stack-up are listed in **Table S1**.

**Table S1:** Target thicknesses of process layers used DUV 248 nm photolithography and I-line (365 nm) photolithography process. The process layers are consistent with the MIT LL SFQ5ee process nomenclature.

| Layer | SFQ5ee (nm) | Reticle | stitching |
| --- | --- | --- | --- |
| M4 | 200 | EX4 | No |
| I4 | 200 | EX4 | No |
| M5 | 135 | EX4 | No |
| I5 | 280 | EX4 | No |
| J5 | 250 | EX4 | No |
| R5 | 40 | EX4 | No |
| C5J | 80 | EX4 | No |
| C5R | 80 | EX4 | No |
| M6 | 200 | I-line | No |
| I6 | 200 | I-line | No |
| M7 | 200 | I-line | yes |
| I7 | 200 | I-line | No |
| M8 | 250 | I-line | No |

We used FIB SEM, STEM, and elemental analysis techniques to characterize the ELASIC. In particular, we characterized the metal-to-metal spacing, quality of interconnects, and dielectric thickness between metal layers. For example, we used STEM EDX (energy dispersive X-ray) to inspect metal layers, oxide-free via formation, junction elements, and anodized Al layers. **Figure S2** shows an enlarged STEM cross-sectional view of the junctions and corresponding elemental analysis of the junction area. STEM EDX was used to identify the individual metal (Nb, Al) and dielectric layers present in the junction. This is particularly important for Al and Nb diffusion during anodization. The bottom-left image in **Figure S2** shows that the formation of the anodized layer extended below the Al layer. We also performed an elemental analysis to confirm clean (oxide-free) via formation.


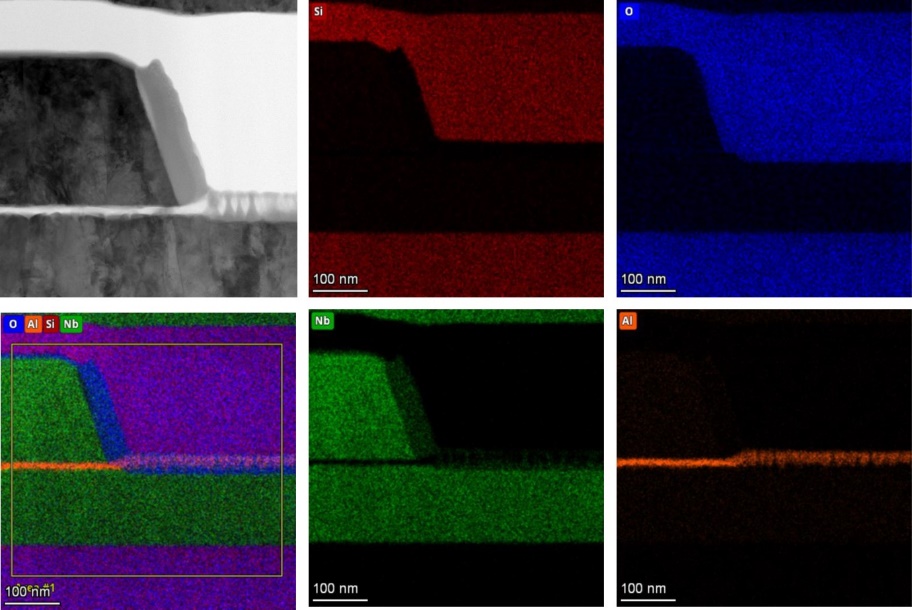


**Figure S2:** A scanning transmission electron microscope (STEM) cross-section image and corresponding energy dispersive X-ray (EDX) images for elemental analysis.





**Figure S3:** SEM of patterned 1.5 µm bump for 5µm pitch flip-chip deposited on under bump metal (UBM) pad of superconducting chip carrier (SSC). The fabrication process with Deep UV (DUV) photomask supports small (5-10 µm) pitch flip-chip interconnects for heterogeneous integration.

A key focus of this study is the introduction of an active JJ layer into the passive chip carrier platform to create an active ELASIC-based chip carrier with functional blocks on a scalable multi-chip platform.

As a first step to evaluate 5 µm pitch micro-bumps, we designed a daisy chain test structure to measure the continuity and critical current of the interconnect. The design used Deep UV (DUV) lithography to fabricate 1.5 µm, 2.0 µm and 2.5 µm diameter bumps. Gold, indium and tin were used for micro-bump fabrication to provide gold-to-gold, gold-to-indium, gold-to-tin, indium-to-indium and indium-to-tin interconnect combinations for flip-chip bonding. **Figure S3** shows a representative SEM image of the daisy chain test structure. The initial flip-chip bonded samples with 1.5 µm gold bumps bonded to 1.5 µm tin (gold-to-tin combination) showed a 5-10 mA critical current of the niobium interconnect. Interconnect combinations, bump diameter, bump height and bump-bonding parameters are under continued investigation to optimize the flip-chip attachment process for improving Nb critical current.

An additional focus of this study was the optimization of the stitch boundary at the overlay between two exposed reticles. To supplement the discussion in the main text, **Figure S4** shows a representative sample of high-magnification SEM images of *0.8* µm line lithography at the stitch boundary in areas of horizontal and vertical overlap.

**
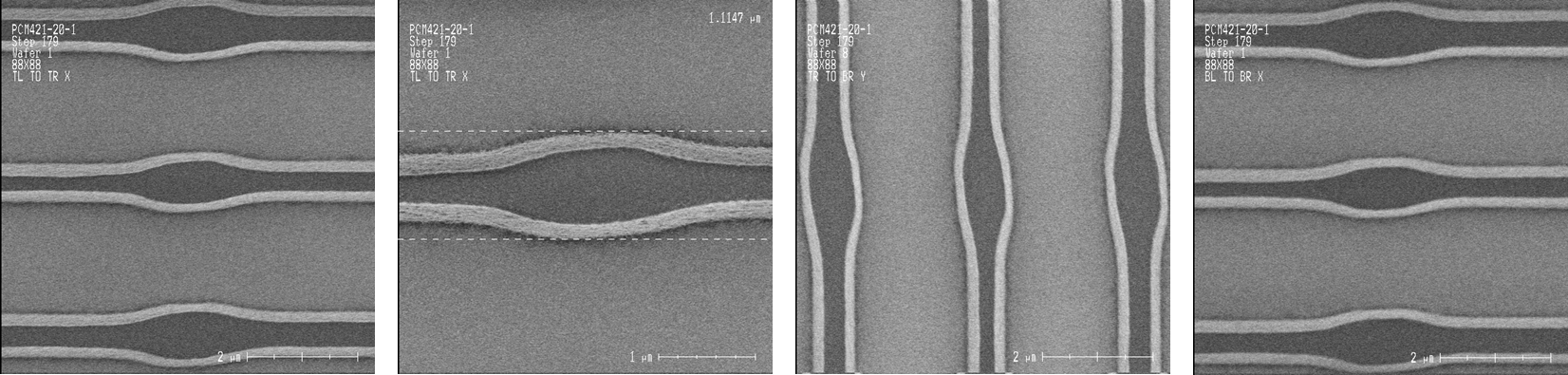
**

**Figure S4:** SEM micrograph of linewidth-compensated 0.8 µm stitched snake & combs lines lithography at the stitch boundary, prior to metal etching. Stitched lines 0.8 µm in width showed linewidth reductions of approximately 70-110 nm within a 1 µm region of overlap. To make the stitching process manufacturable, we impose a design rule for stitched wires with a width of 1.5 µm or narrower to increase the local linewidth by 0.25 µm on each side of the stitch boundary in the 1 µm long overlap region. This additional region of increased linewidth compensates for the post-fabrication reduction at the overlap, in addition to compensating for potential misalignment. The SEM images shown are post-compensation lithographically defined lines prior to metal etching, and include the additional 0.25 µm shapes in the GDS artwork files used to create these stitched lines. SEM images of 0.8 µm wide lines are collected post-lithography as part of the process control monitor test during wafer fabrication.

Flip-chip integration of multiple superconducting chips on superconducting chip carrier will create superconducting multi-chip module (SMCM), **Figure S5** presents a comparison between active and passive chip carriers for chip-to-chip communication.


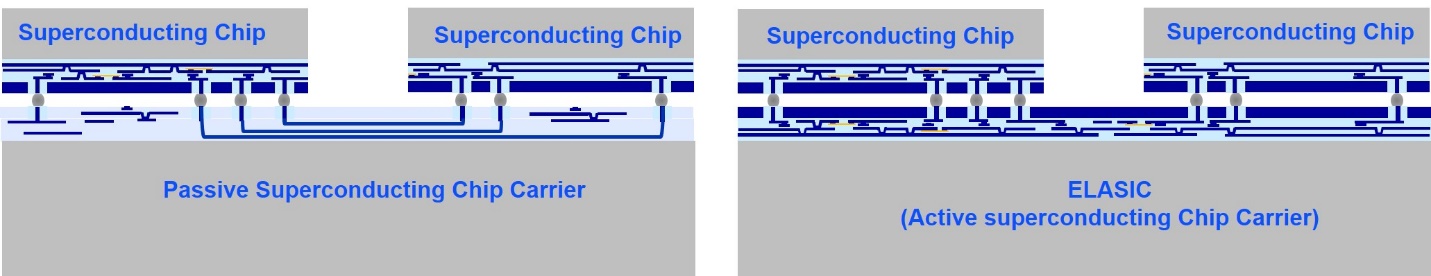


**Figure S5:** Left: Passive superconducting chip carrier, illustrating usage in a flip-chip configuration for chip-to-chip connection (current state of the art). Here passive superconducting chip carrier flip-chip bonded with superconducting chips to create a passive SMCM; Right: ELASIC chip carrier (this work), shown integrated into a flip-chip system. A large format active superconducting chip carrier technology (ELASIC) should feature active and passive superconducting transmission lines, and driver-receiver circuits to distribute information without loss of signal integrity between widely spaced integrated circuits, and the potential for data buffering or memory within the ELASIC.

# **S2: Room temperature measurement data from wafer prober**

We designed various test structures in an ELASIC circuit: JJ repeatability, JJ variability under various metal stack-ups, JJ strings, via chains, snakes, and metal wires. Room-temperature measurements were used for the fabrication process yield, as well as to gauge cross-wafer uniformity. We used an automated wafer probe to measure the junction, via, and wire resistances across each wafer. **Figure S6** shows a room-temperature analysis of a few representative ELASIC circuits.

The parametric variation of R_n_ at the water level is inherently larger than the die level variation owing to contributions from two uncorrelated sources of variation, which is primarily due to photolithography and etching variation in the junction area, while wafer-scale variation is largely due to other factors including focus uniformity and JJ barrier thickness uniformity. Despite this, uniformity on the order of 3% 1σ is not prohibitive even for large scale circuits – typical circuit elements used for memory and communication across SCE logic families, including QFP inverters, PTL drivers and Receivers, JTLs, and shift registers, have much wider margins than logic elements, on the order of +/- 50% tolerance to bias current or J_c_ variation. Despite limited statistics, if a 3% 1σ implies a 6σ variation on the order of 18%, then parametric variation will not limit the integration scale for these classes of circuit elements. Circuits with higher sensitivity to variation may be placed on chiplets to be bonded to the larger carrier, allowing for lower total variation enabled by prescreening of known good dies.


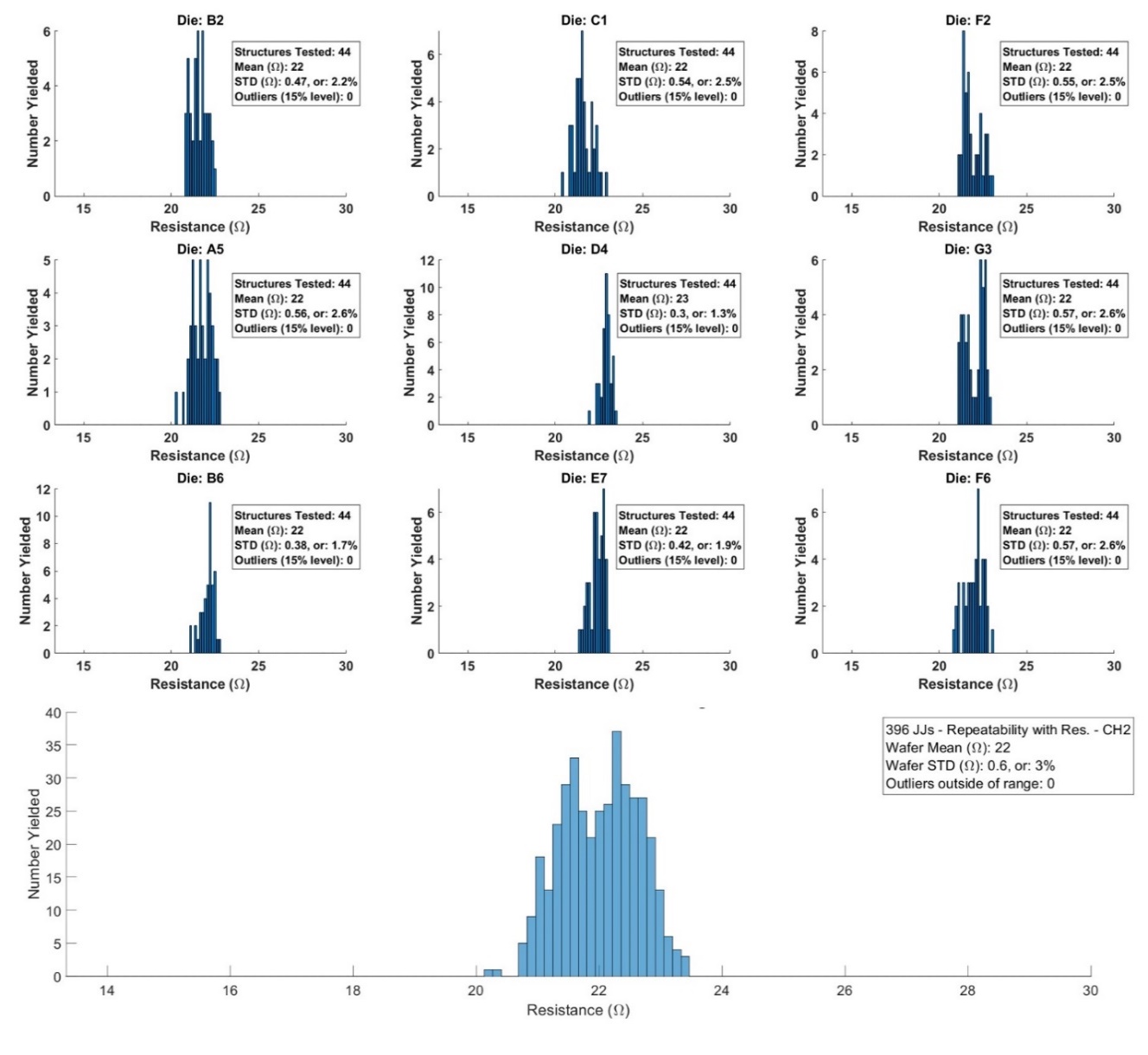


**Figure S6:** Representative results of room temperature electrical testing for an ELASIC wafer using 1000nm Junctions. Histogram of yield for die level as well as wafer level JJ uniformity for ELASIC wafer.
